# Supplementary material for: More meteorological events that drive compound coastal flooding are projected under climate change
Source: Commun Earth Environ. 2020 Nov 12;1(1):47. doi: 10.1038/s43247-020-00044-z (PMC7661409; doi:10.1038/s43247-020-00044-z)
Supplement: Supplementary file 1 — Supplementary Information [file 43247_2020_44_MOESM1_ESM.pdf]

More meteorological events that drive compound coastal flooding are projected under climate change

Emanuele Bevacqua*[1,2], Michalis I. Vousdoukas[3], Giuseppe Zappa[1,4], Kevin Hodges[1], Theodore G. Shepherd[1], Douglas Maraun[2], Lorenzo Mentaschi[3], and Luc Feyen[3]

1. University of Reading, Department of Meteorology, Meteorology Building, Whiteknights Road, Earley Gate, Reading RG6 6ET, United Kingdom.
2. Wegener Center for Climate and Global Change, University of Graz, Brandhofgasse 5, Graz 8010, Austria.
3. European Commission, Joint European Research Centre (JRC), Via Enrico Fermi 2749, Ispra I-21027, Italy.
4. Istituto di Scienze dell'Atmosfera e del Clima, Consiglio Nazionale delle Ricerche (ISAC-CNR), Via Piero Gobetti 101, Bologna 40129, Italy.

*e-mail: e.bevacqua@reading.ac.uk

Supplementary Discussion

Comparison with previous studies

Overall, the results of our present-day assessment based on ERA-Interim reanalysis are in agreement with previous analyses. For example, we find that northern Australia experiences higher probability of concurrent meteorological tide and precipitation extremes than southern Australia, which is likely due to the fact that only the former region regularly experiences tropical cyclones (Fig. 1). This is in agreement with other studies that use field observations[1,2,3] and modelling results[1]. Given that the wind modulates meteorological tides, this is also in agreement with results reported by Martius et al.[4], who studied concurring precipitation and wind high values. In agreement to Wahl et al.[5], who analysed the concurrence of precipitation and storm surge extremes based on station observations, we find a relatively high concurrence probability along the US Gulf and western coasts, consistent with the local high Hurricane activity. With respect to two previous studies, our results indicate slightly higher concurrence probabilities along the southeastern Australia[1] and western US coasts[5], which might be related to the resolution of the ERA-Interim reanalysis used here, which is lower than the data resolution used in these other two mentioned continental studies. In these regions, our results appear more in agreement with the findings of Ward et al.[3], who analysed the co-occurrence of river discharge and storm surge extremes. In some regions, the grid point precipitation from the reanalysis used here could be better correlated with the river discharge (influenced by the precipitation collected over the relatively large catchment area) than with local precipitation from stations (Wahl et al.[5] used precipitation from stations within 25km radius of the tide gauges). In agreement with Ward at al.[3], we also found high concurrence probabilities for the mouth of the short rivers along the coast of Japan. The results appear in agreement also along the southern part of South Africa, though the limited observations render difficult a comparison there. Our findings further confirm previous observations along the European coastline[6,3].

Finally, the spatial variability in large-scale concurrence probabilities found here and in Couasnon et al.[7] is similar and discussed in detail in Bevacqua et al.[8], where a direct comparison between a precipitation and river discharge based assessment is carried out.

Region	$T_{\text{past}}(yrs)$	$\Delta T(\%)$	$\Delta T^{\text{relative}}_{\text{prec.}}(\%)$	$\Delta T^{\text{relative}}_{\text{met. tide}}(\%)$	$\Delta T^{\text{relative}}_{\text{dep.}}(\%)$
Alaska/NW Canada (ALA)	29	-74	-62	-15	23
Amazon (AMZ)	37	>1000	35	11	53
Central America/Mexico (CAM)	15	-22	-68	0	-32
Central Europe (CEU)	12	-50	-90	-9	-1
E Canada/Greenland/Iceland (CGI)	20	-57	-92	6	-2
Central N America (CNA)	6	-22	-72	7	-21
E Africa (EAF)	188	-20	-71	-17	13
E Asia (EAS)	10	-62	-54	-9	-38
E N America (ENA)	7	-43	-70	11	19
S Europe/Med (MED)	12	13	-23	59	-18
N Asia (NAS)	18	-63	-84	8	-9
N Australia (NAU)	7	-16	-34	28	-38
NE Brazil (NEB)	122	189	-16	34	50
N Europe (NEU)	24	-66	-72	-19	9
S Africa (SAF)	14	65	-33	50	17
Sahara (SAH)	112	148	34	3	63
S Asia (SAS)	9	-10	-58	34	-8
S Australia/New Zealand (SAU)	31	-11	-54	7	39
SE S America (SSA)	37	-21	-55	-6	40
SE Asia (SEA)	21	-7	-64	35	-1
W Africa (WAF)	168	-26	-64	-22	14
W Asia (WAS)	114	33	-10	0	90
W Coast S America (WSA)	66	-41	-57	0	43
W N America (WNA)	8	-48	-65	-9	-26
World (WORLD)	17	-20	-77	20	-3

Table 1: **Aggregated statistics of present-day joint return period and associated changes for IPCC subregions and worldwide.** T_{past} is the regional median present-day joint return period of concurrent extremes (based on Fig. 1). $\Delta T(\%)$ is the regional median of the projected change of return periods (based on Fig. 3). $\Delta T^{\text{relative}}_{\text{prec.}}(\%)$, $\Delta T^{\text{relative}}_{\text{met. tide}}(\%)$, and $\Delta T^{\text{relative}}_{\text{dep.}}(\%)$ are the regional relative importance of the projected changes in the three meteorological drivers of compound flooding (precipitation, meteorological tides, and the dependence between precipitation and meteorological tides) (based on Fig. 4). These relative contributions are defined based on the approach used in previous studies[9,10] such that the sum of the individual absolute values add up to 100. Given a region and a driver (i), we first compute the regional median of the return period change caused by the individual three drivers i ($\Delta T_{\text{exp i}}$), and then define $\Delta T^{\text{relative}}_{\text{exp i}}(\%) = 100 \cdot \Delta T_{\text{exp i}}(\%)/(\sum_{i=1}^{3} |\Delta T_{\text{exp i}}(\%)|)$.

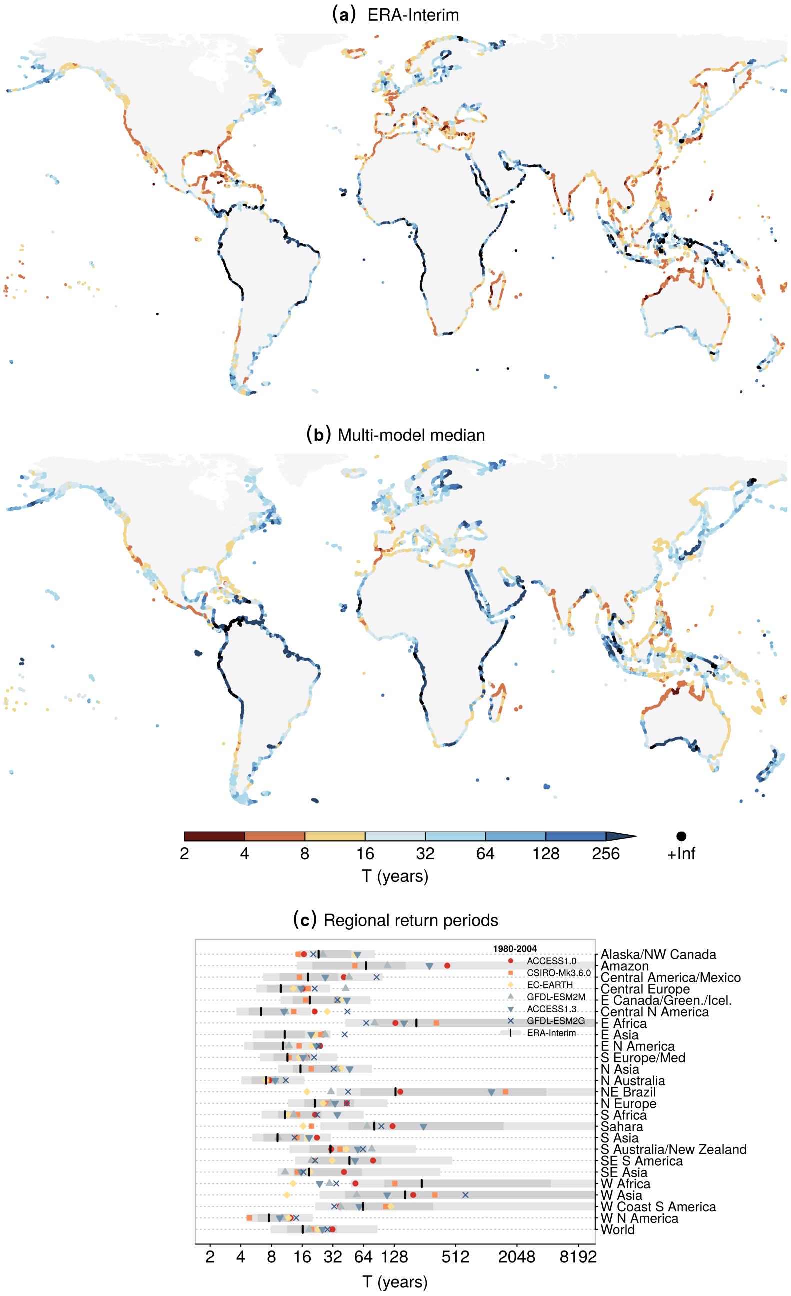

Figure S1: **Comparison between the joint return period of concurrent precipitation and meteorological tide extremes based on ERA-Interim and CMIP5 models.** Joint return periods (inversely probability) of concurrent meteorological tide and precipitation extremes based on **(a)** ERA-Interim and **(b)** CMIP5 models (multimodel median). **(c)** Median value of return periods in IPCC subregions defined in Supplementary Fig. S6 and in the world, based on individual models. Dark and light grey shadings illustrate the ERA-Interim (regional median) 68% and 95% return periods ranges due to natural variability, respectively (e.g., the 95% range is given by the regional medians of the 2.5th and 97.5th percentiles). Return period ranges due to natural variability are computed as explained in the Methods, but with $N_{\text{bootstrap}} = 300$. The return periods are based on the period 1980-2004, i.e. the intersection of the ERA-Interim and CMIP5 models' time domain.

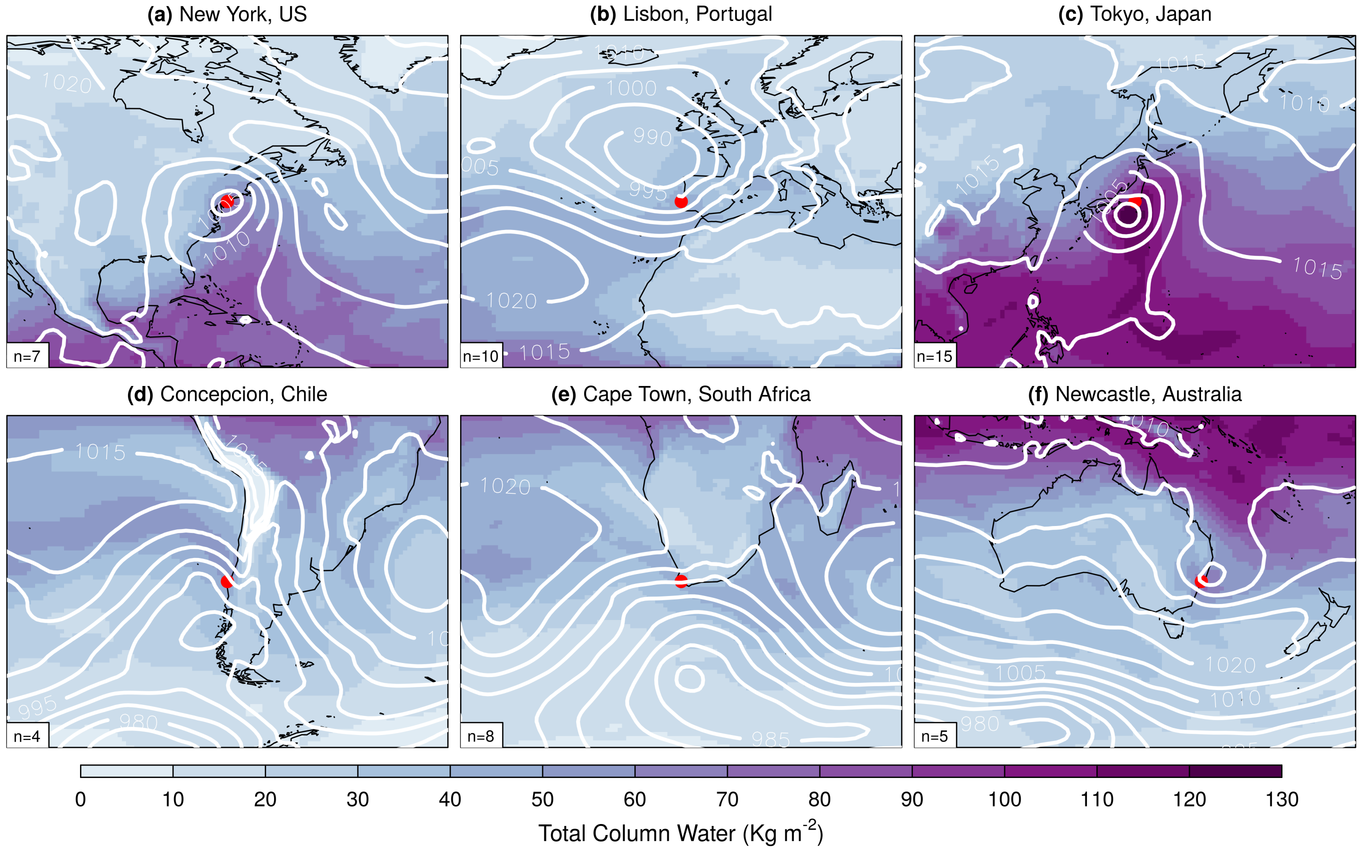

Figure S2: **Synoptic weather conditions driving co-occurring meteorological tide and precipitation extremes.** Composite maps of sea-level pressure (hPa, in white) and total column water fields (shading) for days when co-occurring meteorological tide and precipitation extremes ($> 99.5^{th}$ percentile) occurred in six selected locations. Precipitation time-series are accumulated within a 3-day centred window. The six locations are: **(a)** New York, US[5,11] **(b)** Lisbon, Portugal[12], **(c)** Tokyo, Japan[13,14], **(d)** Concepción, Chile[15], **(e)** Cape Town, South Africa[16,17], and **(f)** Newcastle, Australia[18]. The total number of events considered for computing the composite maps is shown at the bottom-left corner of the panels. The figure is based on ERA-Interim data (1980-2014).

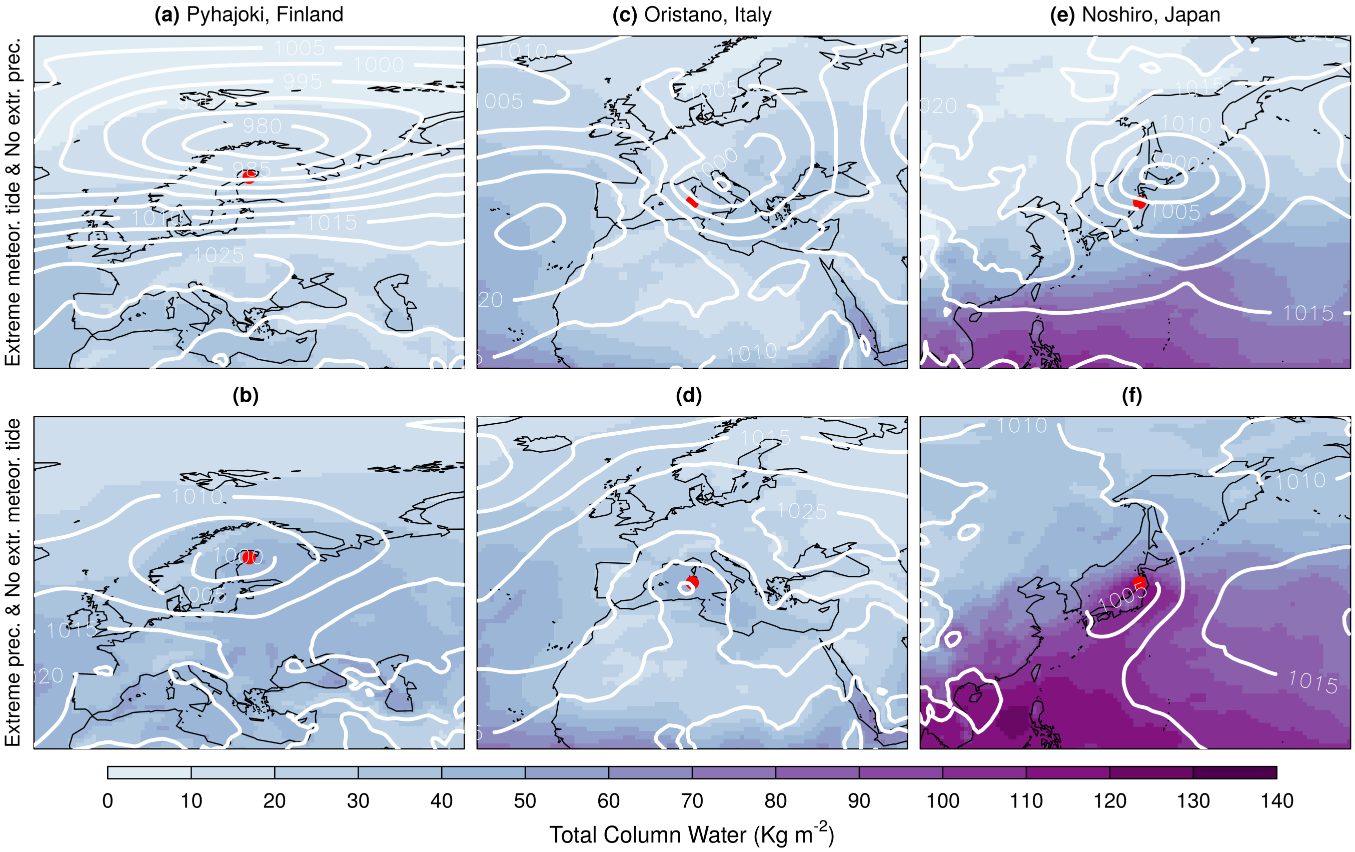

Figure S3: **Synoptic weather conditions driving meteorological tides and extreme precipitation in locations with a low probability of concurrent extremes.** Composite maps of sea-level pressure (hPa, in white) and total column water fields (shading) for days where extremes (> 99.7th percentile) in meteorological tide and precipitation (accumulated within a 3-day centred window) occurred in the nearest grid-point to: **(a-b)** Pyhäjoki, Finland, **(c-d)** Oristano, Italy, **(e-f)** Noshiro, Japan. In these locations, no co-occurring precipitation and meteorological tide extremes occurred in the analysed dataset (1980-2014, based on ERA-Interim data) (no concurrent extremes are found also when defining extremes via the 99.5th percentiles). Further analyses show that the meteorological tide and precipitation extreme events considered in the composites are always driven by distinct weather systems (i.e., weather systems crossing the location not less than 10 days after each other).

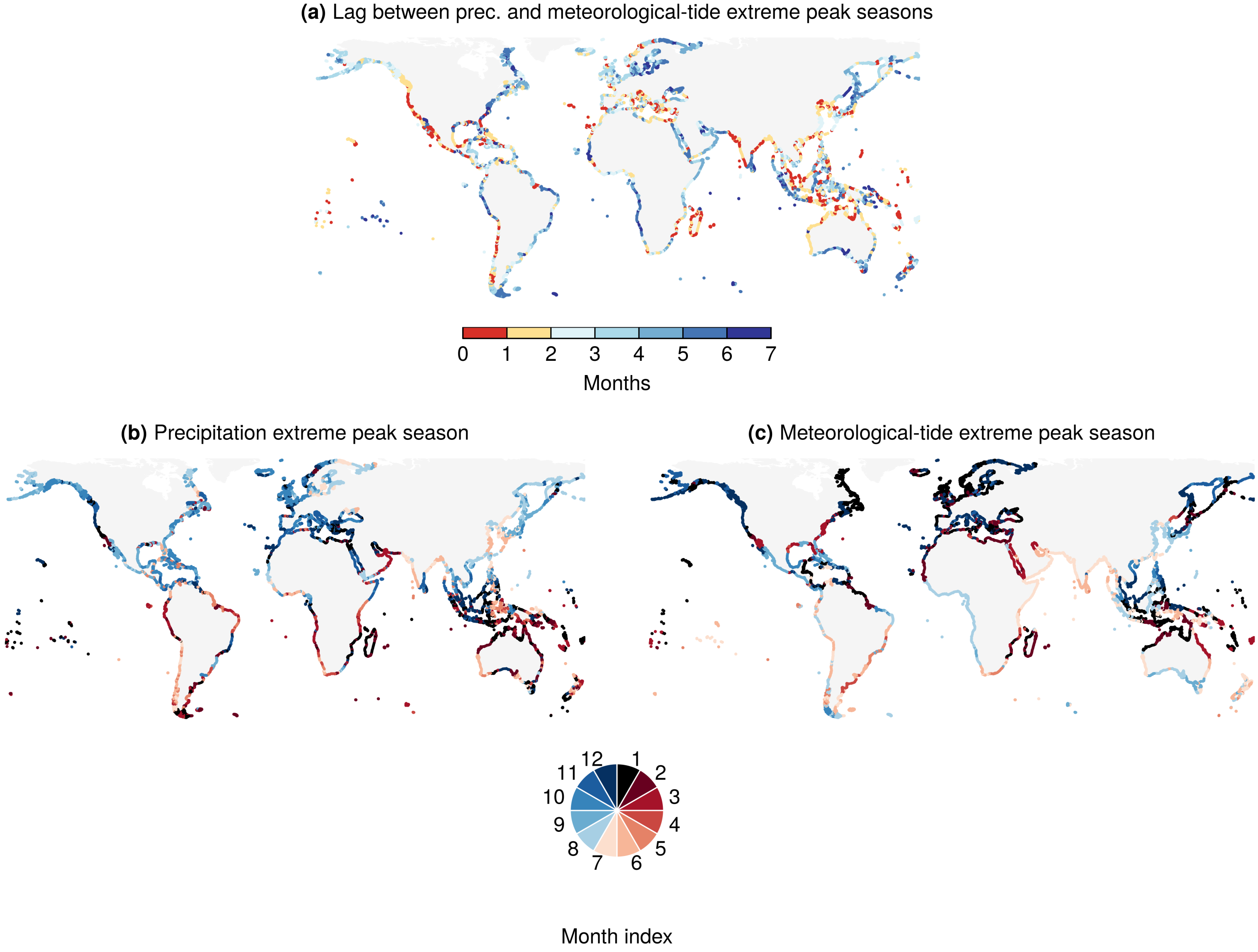

Figure S4: **Seasonality precipitation and meteorological tide extremes.** **(a)** Temporal lag between the peak seasons of meteorological tide and precipitation extremes. Peak seasons are defined as the month of maximum occurrence of precipitation and meteorological tide extremes (values $> 99.5^{\text{th}}$ percentiles), which are shown in panels **(b)** and **(c)**, respectively. Figures are based on the ERA-Interim data (1980-2014).

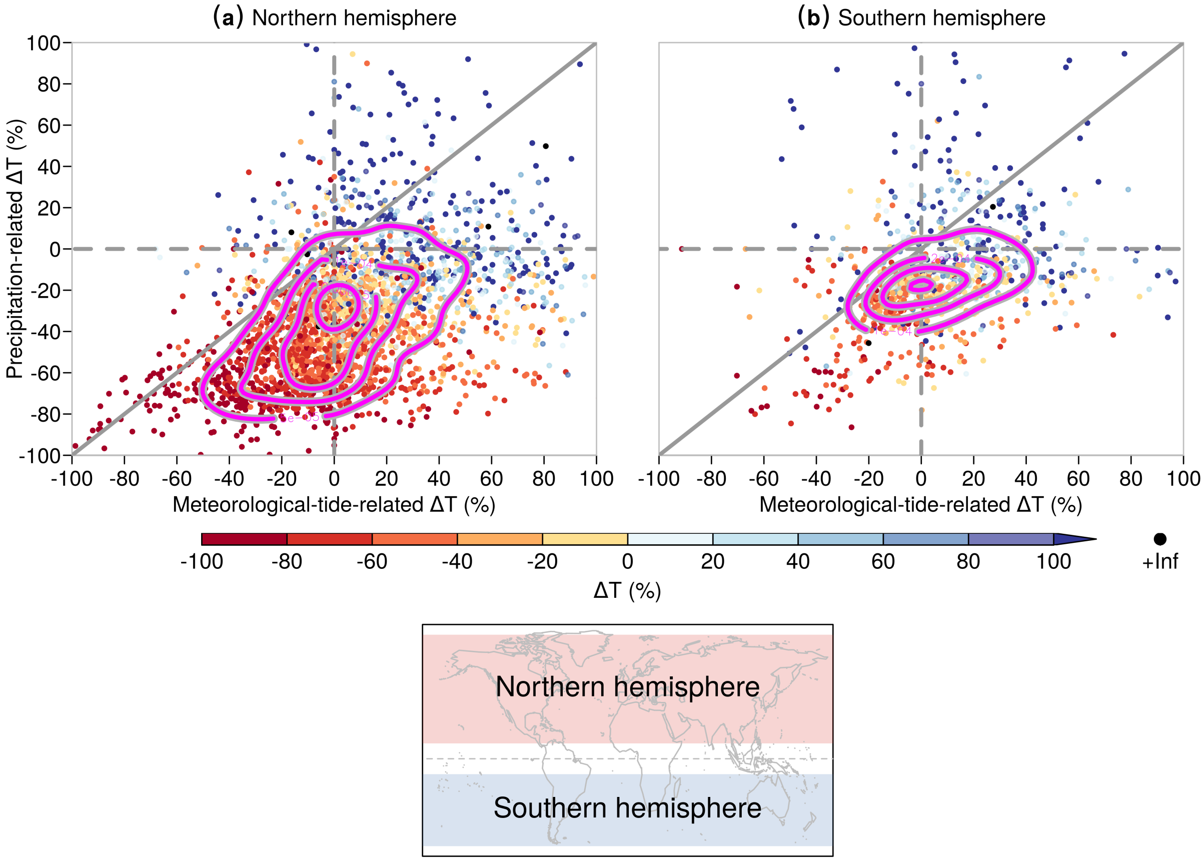

Figure S5: **Relationship between future changes in joint return period driven by meteorological tide and by precipitation.** Scatterplot of the median projected changes (%) in joint return period (inversely probability) of concurrent extremes driven only by changes in precipitation (y-axis, taken from Fig. 4a), or in meteorological tide (x-axis, taken from Fig. 4c). Each dot represents a different location of the **(a)** Northern Hemisphere (light red area in bottom panel), and **(b)** Southern Hemisphere (light blue area in bottom panel). The colour of the dots represents the magnitude of the projected change in joint return periods (%) (taken from Fig. 3a). The magenta contours represent the kernel-based estimation of the probability density function of the Y and X variables in the scatterplots. The part of the tropics lying between $\pm 10^{o}$ is not considered, as cyclones are rare there.

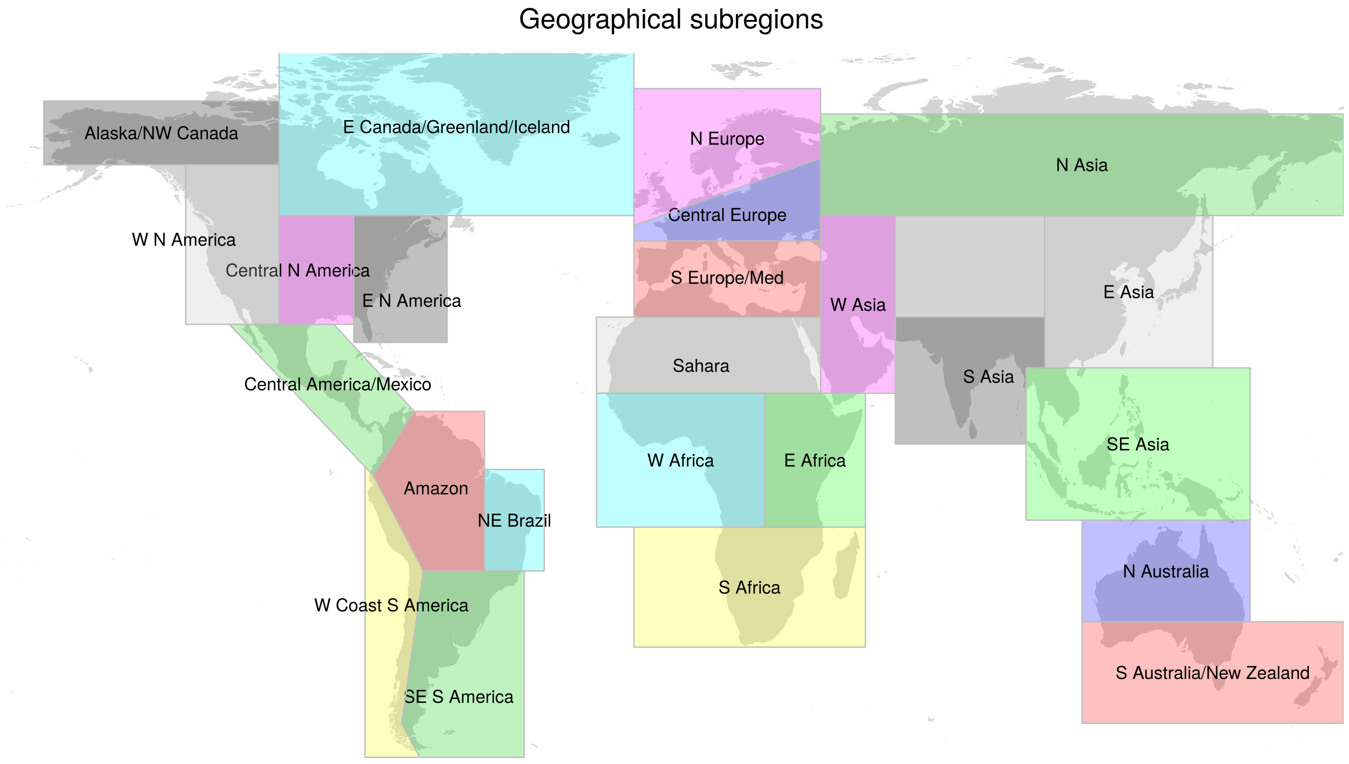

Figure S6: **Geographical regions considered in the study.** The subregions are extracted from those defined in the IPCC SREX report[19] (the coordinates of the regions are available in the report). The "World" region used in Fig. 5 and Supplementary Table 1 includes all the available grid points.

References

[1] Wu, W. *et al.* Mapping dependence between extreme rainfall and storm surge. *Journal of Geophysical Research: Oceans* **123**, 2461–2474 (2018).

[2] Zheng, F., Westra, S. & Sisson, S. A. Quantifying the dependence between extreme rainfall and storm surge in the coastal zone. *Journal of hydrology* **505**, 172–187 (2013).

[3] Ward, P. J. *et al.* Dependence between high sea-level and high river discharge increases flood hazard in global deltas and estuaries. *Environmental Research Letters* **13**, 084012 (2018).

[4] Martius, O., Pfahl, S. & Chevalier, C. A global quantification of compound precipitation and wind extremes. *Geophysical Research Letters* **43**, 7709–7717 (2016).

[5] Wahl, T., Jain, S., Bender, J., Meyers, S. D. & Luther, M. E. Increasing risk of compound flooding from storm surge and rainfall for major us cities. *Nature Climate Change* **5**, 1093–1097 (2015).

[6] Bevacqua, E. *et al.* Higher probability of compound flooding from precipitation and storm surge in europe under anthropogenic climate change. *Science advances* **5**, 10.1126/sciadv.aaw5531 (2019).

[7] Couasnon, A. *et al.* Measuring compound flood potential from river discharge and storm surge extremes at the global scale. *Nat. Hazards Earth Syst. Sci.* **20**, 489–504 (2020).

[8] Bevacqua, E., Vousdoukas, M. I., Shepherd, T. G. & Vrac, M. Brief communication: The role of using precipitation or river discharge data when assessing global coastal compound flooding. *Natural Hazards and Earth System Sciences* **20**, 1765–1782 (2020).

[9] Winsemius, H. C. *et al.* Global drivers of future river flood risk. *Nature Climate Change* **6**, 381–385 (2016).

[10] Vousdoukas, M. I. *et al.* Climatic and socioeconomic controls of future coastal flood risk in europe. *Nature Climate Change* **8**, 776–780 (2018).

[11] Zscheischler, J. *et al.* Future climate risk from compound events. *Nature Climate Change* **8**, 469–477 (2018).

[12] Trigo, R. M. *et al.* The deadliest storm of the 20th century striking portugal: Flood impacts and atmospheric circulation. *Journal of Hydrology* **541**, 597–610 (2016).

[13] Hirano, K. *et al.* Prediction of typhoon storm surge flood in tokyo bay using unstructured model adcirc under global warming scenario. In *ASME 2014 4th Joint US-European Fluids Engineering Division Summer Meeting collocated with the ASME 2014 12th International Conference on Nanochannels, Microchannels, and Minichannels*, V01DT28A008–V01DT28A008 (American Society of Mechanical Engineers, 2014).

[14] Re, S. *Mind the risk: a global ranking of cities under threat from natural disasters* (Swiss Re, 2013).

[15] Rojas, O. *et al.* Flooding in central chile: Implications of tides and sea level increase in the 21st century. *Sustainability* **10**, 4335 (2018).

[16] GWS Global warning system. Storm and flooding in western cape (2017). URL https://www.mynewsdesk.com/se/gws-production-ab/blog_posts/storm-and-flooding-in-western-cape-59153/.

[17] International Federation of Red Cross And Red Crescent Societies. Emergency plan of action final report South Africa: Storm surge (2017). URL https://reliefweb.int/report/south-africa/south-africa-storm-surge-dref-operation-n-mdrza007-final-report.

[18] Savioli, J., Smith, G., Medi, P. & Haines, P. Analysis of extreme ocean water levels at the hunter river entrance (technical report) (2008).

[19] Seneviratne, S. I. *et al.* Changes in climate extremes and their impacts on the natural physical environment, in managing the risks of extreme events and disasters to advance climate change adaptation. a special report of working groups I and II of the intergovernmental panel on climate change (IPCC) **chap. 3**, 109–230 (2012). URL https://doi.org/10.1017/cbo9781139177245.006.
